# Use of Gelatinized Maca (Lepidium Peruvianum) in Early Postmenopausal Women

**Published:** 2005-06

**Authors:** H. O. Meissner, W. Kapczynski, A. Mscisz, J. Lutomski

**Affiliations:** 1 *Faculty of Health Studies, Charles Sturt University & TTD International Pty Ltd, Sydney, Australia;*; 2 *Specialist Gynecology Clinic, 14 Powstancow Wlkp., Poznan, Poland;*; 3 *Research Institute of Medicinal Plants, 27 Libelta St., Poznan, Poland*

**Keywords:** maca (lepidium peruvianum), post menopause, hormones, plant alternative to HRT

## Abstract

**Objective:**

This double-blind, placebo-corrected clinical pilot study was aimed at assessing the use of hypocotyls of cruciferous Andean plant Maca (Lepidium peruvianum Chacon), in alleviating symptoms of menopausal discomfort experienced by women in early post menopause as measured by profiles of serum hormones: Luteinizing Hormone (LH), Follicle-stimulating Hormone (FSH), Estrogen (E2) and Progesterone (PG) and as assessed by Greene’s Menopausal Index.

**Design:**

Study was conducted on 20 Caucasian healthy early-postmenopausal women volunteers during the three months period (Trial I) and on eight women during nine months period (Trial II). Hormone levels were determined in blood with a simultaneous assessment of menopausal index at the start of study, after one month use of placebo, and after two and eight months administration of 2g gelatinized Maca root powder (Maca-GO) in the form of two 500mg hard gel capsules, twice daily.

**Results:**

In comparison to placebo, after both, two and eight months administration of Maca-GO capsules to EPMW, level of FSH significantly (*P*<0.05) decreased with a simultaneous significant (*P*<0.05) increase in the LH level, resulting in significant (*P*<0.05) increase in both E2 and PG, after eight months of Maca-GO treatment only. There was a significant (*P*<0.05) placebo effect resulting in an elevated PG level after one month administration of placebo capsules. Changes in hormone levels was accompanied by substantially-reduced feeling of discomfort associated with menopause, although, there was a distinctive, positive placebo effect as judged by responses to Greene’s questionnaire.

**Conclusions:**

It is reasonable to suggest that Maca-GO when used in EPMW, depending on the length of use, was acting as a toner of hormonal processes as reflected by decrease in FSH and increased LH secretion, which stimulated production of both ovarian hormones, E2 and PG and resulted in a substantial reduction of menopausal discomfort felt by women participating in the study, with a distinctive placebo effect, thus, fully justifying further, more complex study on effectiveness of Maca-GO as a reliable alternative to HRT program.

## INTRODUCTION

Maca (*Lepidium peruvianum* Chacon) is an annual cruciferous root vegetable (same botanical Brassicaceae family as the turnip and broccoli) which grows at 4.000-4,500 m above sea level in the high Andean plateaus of Peru. Traditionally, Maca has been used by native Peruvians as both a food and medicine. People living in the Andes mountains used Maca, since before the time of the Incas for energy, hormone balancing, healthy thyroid functioning, sexual functioning, PMS, menopause, help to maintain healthy bones, tonic for elderly and convalescence. Properties of Maca as food and dietary supplement have been previously reported by Lutomski (1) and therapeutic and medicinal properties were summarized in publication by Chacon (2), Zurowska (3) and number of other technical publications originated mainly in the USA and Peru (4-11). Traditional use of Maca root, for nutritional and putative medicinal purposes, mainly for its adaptogenic and fertility enhancing properties in humans and animals have been in recent years experimentally proven (7-9).

In July 2002 reports were issued by the National Institutes of Health (NIH) in the USA, indicating that the use of combination drugs in hormone replacement therapy (HRT) in healthy menopausal women, increases the risk of invasive breast cancer, heart disease, stroke, and blood clots, outweighing the drugs’ possible health benefits.NIH stopped a large-scale clinical trial on the Prempro (conjugated horse estrogen, made from horse urine) and a synthetic form of progesterone called progestin. Because of the excessive danger to women posed by this drug, NIH recommended the 16,000 participants stop taking the estrogen/progestin drugs immediately. Therefore, the search for safer alternatives to HRT intensified worldwide and in the USA in particular, where estimated six million women, in the past, have selected an option to help relieve menopausal symptoms using HRT.

One of the alternatives to HRT-Maca which contains no plant hormones (unlike soy/genistein and black cohosh), has been presented at the Anti-Aging Medical Conference in 1997 (5). Since then, it has been seen a dramatic increase in the use of this medicinal herb by doctors practicing CAM (Complementary/Alternative Medicine) in the USA (5). For years, Maca has been used successfully by native people of Peru for hormonal imbalances, menstrual irregularities, fertility, and menopausal symptoms, including hot flashes, vaginal dryness, and loss of energy, libido and depression (6). There were indications (12) that Maca can be helpful in reducing discomfort caused by menopausal symptoms and limited case studies on laboratory animals have shown that Maca can be effective for premenstrual syndrome (PMS) as well (13). Results of studies conducted so far, may suggest that action of Maca relies on plant sterols, which act as chemical triggers to help the body itself produce a higher level of hormones appropriate to the age and gender of person taking it (13).

There are a growing number of health care practitioners in the USA who have integrated various herbal therapies into their medical practice and are aware about Maca functional properties as an available alternative to prescription drugs for effective relief of menopausal symptoms (4). In this respect, women may be advised to work with practitioners who can order tests to establish base line hormone levels before starting the Maca therapy, and follow up two months later with a second series of hormone tests to find out if the dose the patient is taking is sufficient and most appropriate to the particular physiological status.However, due to private nature of treatment involved in clinical practice, relevant data have not been freely available in research literature so far.

Therefore, in this pilot study, an attempt has been made to observe a short- and along-term effect of Maca on changes in levels of four sex hormones and in alleviating symptoms of menopausal discomfort on two selected groups of early-postmenopausal women (EPMW) volunteers.

## MATERIALS AND METHODS

### Aim

The aim of this double blind, placebo-controlled study was to determine the effect of the two (2) and eight (8) months of oral administration of capsules containing pre-gelatinized dried and pulverized hypocotyls of Maca (*Lepidium peruvianum* Chacon). In this and subsequent papers in this series, the name *Lepidium peruvianum* Chacon is used as representing the only-known and traditionally-cultivated Maca plant, with its origin linked to Junin plateau in highlands of central Peruvian Andes, from where Dr Gloria Chacon sourced Maca roots for her pioneering work published back in 1961 (2). Her interest and research on this indigenous plant, resulted in its propagation by Peruvian farmers, initially for local market and subsequently extended into larger Maca plantations, which led to commercialization of this plant for international distribution as Peruvian root vegetable with traditionally- and by now, scientifically-acknowledged therapeutic properties. Currently, Maca has been placed on the list of indigenous plants under Peruvian government protection as the Peruvian natural treasure, the fact already acknowledged internationally by the FAO and its traditional status as dietary supplement being reflected in the relevant UE regulation. All the internationally published research on Maca, which followed work of Chacon (2), was conducted on the cultivated Peruvian Maca. However, due to earlier deposition of a specimen labeled Maca, being apparently a wild-growing plant collected in Bolivian Andes, where locally it is known under the common name Maca and given botanical name *Lepidium meyenii* Walpers, many research papers erroneously used scientific name *Lepidium meyenii* as a representation of material - roots originating from cultivated Maca. Therefore, instead of referring to Maca as *L. peruvianum* or synonym *L. meyenii*, as in many articles Maca is scientifically referred to, after inspecting depositions of both plants in Herbarium in Lima, followed by collection and deposition of both specimens in Herbariums of Medicinal plants in Australia and Poland, it has been resolved to adopt in our work the proper term for cultivated Maca as *Lepidium peruvianum* Chacon, being the only plant with historically valid reference and traditionally established grounds to be referred to in scientific work as Peruvian Maca, representing dietary, therapeutic and medicinal characteristics duly referenced in research literature and reflecting experimental work conducted on this plant to date. The plant species is described in details in monographs by Obregon (10) and Chacon (13), as well as in the catalogue of the flowering plants and gymnosperms of Peru (12).

### Maca

Maca roots, harvested and dried on the plantation site in Junin area (Central Andean Region of Peru between 4200 and 4500 m altitude) with an attestation of organic status, were transported to a processing plant at the National Institute of Agricultural Research, National Agricultural University La Molina in Lima (Peru), where it was subjected to a gelatinization process. After cleaning (washing under pressure) and cutting into pieces, dried hypocotyls of Maca were re-hydrated prior to being exposed to short-term elevated pressure under moist conditions (a proprietary extrusion process), followed by drying and pulverizing. Such treatment of Maca, without any chemicals used in the process, resulted in the final powdered product (Maca-GO) achieving increased density, hence higher product weight load in a capsule and through pre-gelatinization of a starch component in the product (not less than 98% according to BRI Laboratory assay), expected to promote its easier digestion. Composition of the pre-gelatinized Maca-GO powder as received directly from the manufacturer in Lima, Peru and verified by the Analytical Laboratory of the Research Institute of Medicinal Plants in Poznan is given in Table 1.

**Table 1:** Composition of Pre-Gelatinized Maca-GO (Lepidium peruvianum Chacon)

No.	Specification	Unit per 100g of product	Pre-Gelatinized Maca Root Powder (Maca-GO)

1	Energy value	kJ kcal	1235 (295)
2	Moisture	g	5.8
3	Ash	g	4.9
4	Crude Protein	g	11.7
5	Ether Extract	g	4.1
6	Carbohydrates Total	g	73.5
7	Available Carbohydrates	g	52.0
8	Dietary Fiber	g	21.5
9	Vitamin C	mg	659.3
10	Thiamine	μg	167.1
11	Calcium	mg	318
12	Phosphorus	mg	352
13	Sodium	mg	52
14	Potassium	mg	1373
15	Glucosinolates as Synigrine	mg	200
16	Unsaponified fraction	% oil fraction	16
17	Campestral	% unsuponified	7.8
18	Sigmasterol	% unsuponified	4.1
19	β-sitosterol	% unsuponified	24.2
20	Arginine	mg	300
21	Gelatinization Index^a^	%	98.5

a Degree of gelatinization of starch obtained as a result of extrusion process. Assay conducted using the method by the BRI Laboratory, Sydney, Australia.

### Subjects

Twenty early postmenopausal Caucasian women volunteers (Trial I) and eight (Trial II) aged between 45-62 years were selected to this pilot study. They represented healthy EPMW who had experienced their last menstruation not less than 2 months and not more than 12 months prior to the start of the Trial. Prior to the study they were not used any hormonal treatment and were not taking any medications during the study period. A signed informed consent was obtained from all subjects regarding their voluntary participation in the trial conducted under specialist Gynecologist’s supervision in a Private Clinic in Poznan (Poland). All enrolled subjects were informed of the purpose, benefit and possible risks of the study.

### Experimental protocol

Hard gel capsules (size “0”) both placebo and Maca-GO (500mg net per capsule), used in this study were custom-made at the Institute of Medicinal Plants in Poznan. During the 1st month period (Placebo phase), all EPMW received 4 × 500 mg capsules daily of placebo (2,000 mg sorbitol & cellulose daily), followed by the identical dosage of capsules containing Maca-GO (Maca-GO phase). Although Chacon (2, 13) recommends approximately 5 g of Maca powder daily when used as a source of functional food supplementing daily diet, the choice of 2 g/day dose of Maca-GO adopted in this study was based on clinical and practical experience reported by Muller (5, 6), who recommended for women in the USA, to administer 2g of Maca powder per day for alleviation of menopausal discomfort. Capsules were self-administered according to the following schedule: 2 capsules some 30 minutes before the morning and 2 capsules before the evening meal for the period of either one month Placebo followed by two months (Trial I) or eight months (Trial II) of Maca-GO.With the start and at the end of each phase of the trial, all women were interviewed by the gynecologist and requested to answer a set of standard questions according to Greene’s questionnaire. This in order to determine a Menopausal Index at the time when blood was taken for hormone analyses (FSH, LH, E2 and PG).

The study was carried out by specialist Gynecologist and researchers of the Research Institute of Medicinal Plants in Poznan under international supervision between May 2002 and March 2003.

### Assays

Serum LH, FSH, E2 and PG were measured before the Trial (Sampling A), after 1 month placebo treatment (Sampling B) and then, two months (Trial I) or two and eight months (Trial II) after administration of Maca. Hormone assays were conducted by a Clinical Diagnostic Laboratory LABO-MED in Poznan using officially accepted standard chemiluminescence procedure on Immulite-DPC equipment. Precision of this technique is monitored by National Center of Quality of Diagnostic Medical Laboratories in Poland and the Laboratory is a participant of the International Quality Control RIQAS maintained by Randox Company.

### Statistical analysis

Data were expressed as mean (± SEM) where applicable. Statistical analysis was performed by the Student's t-test with the difference considered significant at *P*<0.05.

## RESULTS

### Trial I

The Trial I was started with 20 subjects out of which during the three months duration, eight subjects have failed to conclude the study, either due to sickness (2 women) or due to variety of personal reasons-such as long period of not taking the Maca-GO capsules while on holidays (2 women) or inconsistent manner of their administration (4 women). Therefore, they were excluded from the final summary of results.

Results from hormone assays summarized in Table 2 demonstrate that in comparison to placebo, after two months of using Maca-GO capsules, level of LH significantly increased (*P*<0.05), with statistically non-significant (*P*>0.05) decrease in FSH. On the other hand, one month placebo treatment resulted in a significant (*P*<0.05) increase of blood Progesterone level with a significant (*P*<0.05) reduction observed after two months of Maca-GO administration, with the level falling non-significantly (*P*>0.05) below the level recorded at the start of the Trial I. There were no significant changes observed in the E2 level during the Trial I.

**Table 2:** TRIAL I: Hormone levels in postmenopausal women (n=13) at start of the trial, after one month use of placebo and after two months of Maca-GO treatment (2000 mg/day) by 12 EPMW

Hormone^c^	Start of the Trial	Post-30d Placebo	Post 2m Maca-GO

FSH (mIU/mL) ± SE^d^	2 56.4 ± 1.03^a^	57.2 ± 1.85^a^	48.8 ± 2.32^a^
LH (mIU/mL)	24.4 ± 0.66^a^	26.6 ± 0.71^a^	31.7 ± 0.86^b^
PG (ng/mL)	0.45 ± 0.07^a^	0.72 ± 0.09^b^	0.33 ± 0.08^a^
E2 (pg/mL)	25.6 ± 4.21^a^	25.2 ± 3.83^a^	26.1 ± 3.96^a^

c Values in a row with unlike letters indicate significant difference at P<0.05;

d ± SE = Standard Error of Mean.

In personal interviews conducted by a gynecologist during the Trial I, as many as 42% participating women have reported reduction in feeling of menopausal discomfort already after one month administration of Placebo capsules. According to subjective assessment by participants, after two months of Maca-Go treatment, majority of women (9 of 12 which concluded the three month trial) observed noticeable reduction in feeling discomfort, typical to menopause, which they experienced at the start of the Trial. However, according to responses given by participants in Greene’s questionnaire (Table 3), after one month of Placebo application, they already reported improvement in such characteristics as: reduced nervousness, less difficulties in falling asleep, better concentration improved ability to concentrate, feeling of being more energetic, less frequent feeling of numbness and headaches as well as a reduction in night sweating. After further two months of Maca-GO application, correcting responses for Placebo effect, there was a positive reaction of EPMW to the following symptoms: an increase in feeling of being more energetic, lesser nervous tension, reduction in hot flushes and return of interest in sex life, as assessed according to the Greene’s menopausal index. In symptoms covered by other questions, and in particular incidence in night sweating, feeling of anxiety, state of depression, excessive crying, irritability, and the responses were inconclusive.

**Table 3:** TRIAL I: Summary of Greene’s Menopausal Test Score^a^ after 30 days placebo (B) and after two months of use of encapsulated Maca-GO (2,000mg/day) by 12 EPMW

Question No	Symptom (according to Greene’s Test)	Score after 1m Placebo expressed as % of the evel at start of the Trial (placebo effect)	Score after 2m use of Maca-GO expressed as % of the Placebo score(placebo-corrected)

1	Abnormally-fast heart beat rate	75.0	105.9
2	Nervousness	86.8	103.1
3	Difficulties in falling asleep	83.9	104.0
4	Excessive alertness	93.9	96.9
5	Sudden feeling of anxiety	109.1	126.3
6	Difficulties in concentration	80.6	92.6
7	Feeling of being tired/luck of energy	79.4	87.1
8	Lack of interest	88.0	88.0
9	Unhappiness/state of depression	100.0	104.2
10	Excessive crying	91.7	104.8
11	Irritability	90.9	103.4
12	Loss of consciousness	93.8	107.1
13	Nervous tension	86.7	89.7
14	Numbness/“pins & needles”	72.0	90.0
15	Headaches	84.0	100.0
16	Muscle aces and joints aces	75.8	96.2
17	Loss of feel in feet & hands	94.1	94.1
18	Difficulties in breathing	105.6	111.8
19	Hot flushes	90.3	90.3
20	Excessive night sweating	78.8	104.0
21	Loss of interest in sex life	103.8	93.1
	Average of the total Green’s score	88	89

a Scoring index:1=symptom not experienced; 2 = occasionally; 3 = often; 4 = very often.

### Trial II

Eight women, who volunteered to nine months participation in the study, in which after one month Placebo phase, they were given Maca-GO capsules for eight months period, following the same experimental protocol as in Trial I, all of them concluded the eight month study period.

Results from hormone assays summarized in Table 4 demonstrate that in comparison to placebo, after 2 months of using Maca-GO capsules, level of FSH significantly decreased (*P*<0.05) with further reduction after 8 month of Maca-GO administration. On the other hand, there was a statistically significant (*P*>0.05) increase in LH level after 2 months of Maca-GO intake with a further slight increase after 8 months of administration. Levels of Progesterone increased with progression of the Trial II, but only the value recorded after 8 months of Maca-GO administration was statistically significant (*P*<0.05) as compared to the previous samplings (at the start of the Trial, after placebo and after two months Maca-GO treatment). While there were no significant (*P*>0.05) changes in the E2 levels recorded between the start of the Trial and one month Placebo treatment, the levels recorded after two and eight months of Maca-GO administration were significantly higher (*P*<0.05) as compared to the start of the trial and one month placebo treatment.

**Table 4:** TRIAL II:Hormone levels in postmenopausal women (n=8) at start of the trial, after one month placebo, after 2 month Maca-GO and after 8 month Maca-GO treatment (2,000mg/day) by eight EPMW

Hormone^c^	Start of the Trial	After Placebo (1 month)	Maca-GO (2 months)	Maca-G (8 months)

FSH (mIU/mL) ± SE^d^	54.3 ± 1.22^a^	59.7 ± 1.35^a^	47.3 ± 0.93^b^	39.3 ± 0.90^b^
LH (mIU/mL)	21.5 ± 0.59^a^	23.3 ± 0.68^a^	30.6 ± 0.76^b^	32.9 ± 0.83^b^
PG (ng/mL)	0.41 ± 0.11^a^	0.54 ± 0.14^a^	0.59 ± 0.12^a^	0.78 ± 0.09^b^
E2 (pg/mL)	32.9 ± 3.72^ab^	27.2 ± 2.96^a^	32.0 ± 2.67^ab^	35.7 ± 2.09^b^

c Values in a row with unlike letters indicate significant difference at P<0.05;

d ± SE = Standard Error of Mean.

Subjective assessment by participants of their condition of “wellbeing” during and after completion of the Trial II, as expressed during interviews with the doctor, indicated that all participating women reported overall improvement in reduction of feeling menopausal discomfort experienced at the start of the Trial, with an improvement observed already after one month of placebo treatment.

Responses given by participants to Greene’s questionnaire and expressed as a score of points assigned to each individual question according to severity of symptoms experienced during one week prior to the interviews with the gynecologist (who has recorded all the answers during the trial), confirmed distinctive placebo effect on Greene’s score index (Table 5). All reported responses to individual questions in Table 5 and relevant scores recorded with the end of the two and eight months of Maca-GO application have been corrected for placebo effect (expressed as a percentage of a Placebo score). After two months of Maca-GO application, a noticeable improvement was observed in such characteristics as: feeling of being more energetic and lesser muscle and joints aces less frequent feeling of numbness and headaches as well as a reduction in night sweating.

**Table 5:** TRIAL II: Summary of Greene’s Menopausal Test Score^a^ after 30 days placebo and after 2 and 8 month of use of encapsulated Maca-GO (2000mg/day) by eight EPMW

Question No	Symptom (according to Greene’s Test)	Score after 1m use of Placebo expressed as % of the score at start of the Trial (placebo effect)	Score after 2m use of Maca-GO expressed as % of the Placebo score(placebo-corrected)	Score after 2m use of Maca-GO expressed as % of the Placebo score (placebo-corrected)

1	Abnormally-fast heart beat rate	91	108	78
2	Nervousness	89	101	95
3	Difficulties in falling asleep	89	109	88
4	Excessive alertness	99	94	81
5	Sudden feeling of anxiety	107	103	55
6	Difficulties in concentration	88	94	98
7	Feeling of being tired/luck of Energy	94	88	84
8	Lack of interest	88	97	103
9	Unhappiness/state of depression	91	105	94
10	Excessive crying	97	98	95
11	Irritability	93	96	81
12	Loss of consciousness	98	111	103
13	Nervous tension	87	98	92
14	Numbness/“pins & needles”	84	94	98
15	Headaches	93	97	83
16	Muscle aces and joints aces	78	92	90
17	Loss of feel in feet & hands	97	95	87
18	Difficulties in breathing	106	94	91
19	Hot flushes	95	94	82
20	Excessive night sweating	86	97	86
21	Loss of interest in sex life	107	96	95
	Average of the total Green’s score	93	98	89

a Scoring index:1 = symptom not experienced; 2 = occasionally; 3 = often; 4 = very often.

Eight months of self-administration of Maca-GO capsules by early postmenopausal women, resulted in a distinctive beneficial effect on majority of menopausal symptoms listed in the Greene’s menopausal index with the most pronounced reduction recorded in frequency of sudden feeling of anxiety. Also such characteristics as less incidences of excessive alertness, abnormally-fast heart beat rate, lesser nervous tension, nervousness, irritability and excessive crying, less frequent headaches, unhappiness and being depressed, lesser incidence in muscle and joints aces were distinctively noticed by the participants. This was associated with reduction in a frequency of hot flushes and excessive night sweating with a simultaneous noticeable improvement in falling asleep, feeling to be less tired and more energetic with return of interest in sex life.

In general, accumulated score of responses to Greene’s questionnaire confirmed the fact that after one month placebo, the EPMW reported a 59% reduction in severity of menopausal discomfort, from “occasional” and “often” to “symptoms not experienced” category (Table 6). While further two months of Maca GO treatment has only slightly higher incidence of increasing number of questions assessed in “symptoms not experienced category” then after 8 month of use of Maca-GO, there were twice as much symptoms recorded by women in this category as compared to the score as recorded at the start of the trial. This may be the closest to a single objective numeric representation of a positive effect, which Maca-GO had in this study on participating EPMW in terms of reduction in severity of their menopausal discomfort along the period of the eight months study.

**Table 6:** TRIAL II:Accumulated score in answers to questions reflecting Individual symptoms in Greene’s Menopausal Index^a^

Interview Period	Severity of Symptom (Score)^a^			
	1	2	3	4

Start of the Trial	29.4	38.9	20.0	12.3
After one month Placebo	46.8	25.4	15.5	12.0
After 2 months Maca-GO treatment	49.1	25.8	10.8	14.3
After 8 months Maca-GO treatment	59.5	27.0	7.9	7.1

a Scoring index used in summarizing scores obtained in answers to individual questions in Greene’s questionnaire: 1 = symptom not experienced; 2 = occasionally; 3 = often; 4 = very often.

## DISCUSSIONS

In the pioneering work on Maca by Chacon (2), she established that there were four alkaloids in the Maca root, and not its plant hormones, that produced fertility effects on the ovaries and testes of the rats. These effects were measurable within 72 hours of dosing the animals. She deduced that the alkaloids were acting on the hypothalamus-pituitary gland, which explains why both male and female rats were afflicted in a gender-appropriate manner. This may also explain why the effects of Maca in humans are not limited to ovaries and testes, but through acting on the adrenals, gives a feeling of greater energy and vitality and through this possibly affecting the pancreas and thyroid as well.

Medicinal properties and energizing functionality of Maca for men and women are linked to a peculiar composition of this plant, cultivated and grown in the central Andes of Peru in the puna, where only alpine grasses and native high frost tolerant plants can survive and produce crops of any economic significance. Maca roots, grown and then dried under such a harsh environmental conditions, exposed to full spectrum of solar radiation, low humidity and extreme temperatures between day and night as experienced at such a high elevation, exhibit specific - yet unresolved to the present days - endocrine effect, which, as demonstrated in this paper, helped to alleviate menopausal symptoms in women -as a natural, non-hormonal alternative to the currently used HRT treatment. A superior nutritional quality of Maca (22) and doses as dietary component for use at the level 5g/day as recommended by Chacon (2, 13), may not explain the effects of 2g/day doses administered to EPMW in the Trial I and II. It is most likely that the complexity of components present in Maca root powder such as sterols (campesterol, stigmasterol and beta-sitosterol), polyunsaturated acids and their amides, called “macaenes” and “macamides”(9), aromatic glucosinolates (23) and several alkaloids -yet to be characterized, through their complex synergistic and/or interactive action amongst them, will eventually provide an answer to physiological action of specific doses of Maca recommended for prophylactic and/or specific therapeutic effect for men and women. Dini (22) suggests that observed aphrodisiac powers of Maca for men and women may be ascribed to presence of prostaglandins and sterols in the hypocotyls of Maca and overall fertility enhancing properties may be attributed to the presence of biologically-active aromatic isothiocyanates derived by hydrolysis of the glucosinolates and specifically due to benzyl isothiocyanate and p-methoxybenzyl isothiocyanates (23). On the other hand, benzyl isothiocyanate present in Maca root has been reported to be a potent cancer inhibitor of mammary gland and stomach (24).

Since introduction of Maca to medical practice in the USA, it has been increasingly used to treat menopausal patients at various clinics, where, as reported by Muller (6), some 50 percent of patients were satisfied from using Maca preparations instead of genistein supplements made from soy, which have been shown as having stimulating effect on breast cancer cells (6). Various personalized programs with the use of Maca were developed for patients, some of them using Maca in conjunction with nutritional supplements, to wean women off of hormone replacement therapy. For those women who still had some symptoms, a combination protocols have been developed, with involvement of Maca extract and a minute amounts of natural estrogen together with natural progesterone, which, unlike progestin, is considered not carcinogenic (6). Maca, which has been confirmed, since the first report of Chacon (2), to not contain plant estrogens or any other hormones, through plant sterols, stimulate endocrine system helping to maintain hormonal balance in a way that is not yet well understood (6, 13). These sterols are used by the body with the help of the pituitary to improve adrenal function, ovarian and testicular function, as well as the functioning of the thyroid and the pancreas, and the pineal gland (which makes melatonin). Multi-functional effect of Maca on endocrine relationships may also explain reported in the literature, its positive influence on stimulation of endocrine glands in regulation of hormonal balances in the body in distinctively different way than those reported when women treat menopausal complaints with cimicifuga (16, 17) or preparations based on red clover (18, 19) or soy (20) phyto-estrogens.

It was reasonable to assume, that, as an adaptogenic herb, after two months use of Maca-GO by EPMW, may help to correct symptoms of FSH dominance and may slow down or counteract a decline in E2 level helping in relief from various symptoms and associated manifestations of menopausal discomfort. Extending period of Maca-GO administration to 8 months (Trial II), it was expected to observe differences, if any, in short- and longer-term responses of EPMW to Maca administration in hormone levels and Greene’s Menopausal Index.

Results reported in this pilot study supported both above assumptions, since, in addition to overall relief from most of the menopausal symptoms as subjectively assessed in both Trials and confirmed in answers by participating EPMW to Greene’s questionnaire, the levels of FSH were reduced significantly (*P*<0.05) after both, short (2 months) and longer period (8 months) of Maca-GO administration, although, in Trial I, reduction was substantial but not significant (*P*>0.05). On the other hand, in Trial II, Maca-GO treatment resulted in significant (*P*<0.05) increase in the E2 level after long-term (8 months) of Maca-GO use only, in relation to level recorded after placebo treatment, while there was no significant effect of two months Maca-GO administration.

Observed in this study positive effect of Maca-GO in lowering FSH level after both short- and longer-term use was associated with a parallel significant (*P*<0.05) increase in LH concentration. The ratio between the two hormones (FSH and LH) were maintained in both Trials at the level close to 1.6 after two month of Maca-GO intake and reduced to 1.2 after 8 months of Maca-GO administration, which is typical to commonly observed in early menopausal stage (*P*>1.1).

While in Trial I, two months administration of Maca-GO had no significant (*P*>0.05) effect on PG blood concentration, there was a significant (*P*<0.05) increase in PG after one month placebo intake only, indicating that two months Maca-GO treatment has no effect on changes in PG levels - the fact confirmed by results obtained in Trial II. However, there was a significant (*P*<0.05) increase in progesterone blood concentration after a long-term administration of Maca-GO to EPMW.

Implications of the pituitary stimulating effects of Maca-GO as may be deducted by reduction of FSH and an increase in LH blood concentration are important in this respect, that opens a new avenue to interpretation of the effect of Maca on a complex of physiological processes which may lead to further investigation of possible use of Maca-GO as a “Non-Hormonal Replacement Therapy” – which appears to be a safer and less controversial treatment to currently disputed conventional HRT with the use of pharmaceutical hormone preparations.

The E2 levels observed in women participating in this study were below 30pg/ml in Trial I and between 30 and 35pg/ml levels, which may confirm postmenopausal status of participants and/or dysfunction or atrophy of ovaries. Observations made by Malespina (11), indicated levels of 30pg/ml and above as the absolute minimum that a woman needs to avoid symptoms of discomfort characteristic to post menopause and 60-75pg/ml are considered as adequate levels.

On the basis of results obtained in this study on EPMW, it is reasonable to assume that Maca-GO significantly influenced the organs of internal secretion. By stimulating the pituitary to significant increase in FSH and LH secretion, Maca-GO seemed to contributed to regulatory mechanism responsible for the remaining ovarian functions, which resulted in an increased secretion of the minimum quantity of Estrogen, known to be necessary to avoid or substantially reduce problems of mental, physiological and physical discomfort associated with menopause as manifested by symptoms listed in Greene’s Menopausal Index. Suggestion of Chacon (3, 13), that Maca, through stimulation of pituitary gland, may have indirect effect on pineal gland, which secretes melatonin could be supported by observations made in this study, explaining a sedative and calming effect of Maca-GO on menopausal women, according to symptoms listed in Greene’s Menopausal Index.

Although, positive effect of both, short- and longer-term administration of Maca-GO in alleviating symptoms of menopausal discomfort experienced by EPMW prior to involvement in the study can not be disputed after being confirmed by figures summarized in the Greene’s Menopausal Index, the statistically-significant (*P*<0.05) different response of women in levels of sex hormones recorded after short- and a longer-term use of Maca-GO, may indicate different mechanisms of action of Maca constituents on endocrine system in EPMW - the fact warranting further, more complex study.

The question remains however, whether Maca as adaptogenic herb has a “stimulating” or a “balancing” effect? According to Muller (6) depending on the dosage involved, Maca can have balancing or stimulating effect under wide range of circumstances. In the USA, women with menopausal symptoms are advised to take 3-4 Maca capsules daily for balancing effect, but very sensitive menopausal woman may only need 2 capsules daily. Such a dosage may reduce or completely eliminate hot flashes in as little time as 4 days to a week (6). On the other hand, with too high dosage, in women highly sensitive to Maca, there may be a stimulating and not balancing effect, which actually may increase the amount of hot flashes.

Various commercial Maca preparations are available on the therapeutic market in the USA and Europe and are recommended by practitioners, who, based on their personal clinical experience and limited research conducted so far, recommend Maca to be used by women for number of reasons such as hormone balance, for energy, for PMS, for menopausal symptoms, including hot flashes, vaginal dryness, the “blues”, for thyroid health, immune balance, and nutritional support for postmenopausal women to maintain healthy bones (5, 6, 11). As far as nutritional support to Maca-Go preventive and/or therapeutic treatment of menopausal symptoms or menopausal irregularities concerns, dietary routine in this period of time should be based on the “new food pyramid” based on U.S. Department of Agriculture dietary recommendations released in January 2005, with the prime direction to adopt into daily dietary routine being a strong emphasis on grains, fruits, and vegetables with limited amounts of meats, oils, and fat. The main thrust of the retooled pyramid is “first and foremost, moderation-with necessity to pay attention to what it is eaten, maintaining moderation, and then, being involved in some sort of physical activity -exercise, with the recommendation that even a little practically possible exercise “will make a difference”. There was no requirement for participants to follow up or report on their dietary habits - which may have a distinctive influence on responses of EPMW to the Maca-GO. Therefore, slight discrepancies and variation in hormonal profiles and related subjective assessment of improvement reported by the participating women after two months of Maca treatment in the Trial I and II, may also be attributed to the un-accounted in this study, dietary factor.

Results from the study presented here shows that Maca-GO has a potential to be used in early post-menopausal women and under dosage conditions as described in this paper, through stimulation of secretion of pituitary hormones (FSH and LH) may act on ovaries in a specific manner which induce hormonal balance in this period of woman life, which provides a certain degree of alleviation in menopausal discomfort as expressed in symptoms listed in the Greene’s Menopausal Index. As demonstrated in this study, it is important however, to take into account a strong placebo effect on EPMW involved in assessment of effectiveness of Maca-GO used for treatment of menopausal symptoms.

Analyzing Placebo-corrected score on the list of symptoms in the Greene’s Menopausal Index, both in Trial I and II, it appears that in majority of participating EPMW, after two months of Maca-GO treatment, there was a distinctive reduction in severity of stress related symptoms contributing to overall alleviation in menopausal discomfort. This trend was continued further with an extension of Maca-GO administration into the eight month period (Table 6). In recently-published work by Lopez-Fando *et al*. (21), it was demonstrated, that under laboratory conditions, where a methanolic extract of Lepidium Peruvianum was used, it was possible to attenuate or even eliminate variations in homeostasis produced by stress, with the most visible manifestation being a reduction or even healing of stress-induced ulcers and normalizing corticosterone and glucose levels as well as bringing to normal an increased weight of adrenal glands induced by stress. The question remains however, to what degree results from the use of Maca extract, representing fractions of selected components eluted from the root by methanol or other solvent(s), may be compared with those obtained using gelatinized Maca root powder representing its unaltered therapeutic characteristics in its traditionally-understood coherent complexity of the root preparation and administered at the appropriate dose, specific to the purpose of its use as dietary supplement and/or phyto-therapeutic for cure and/or prevention.

Obtained results and available literature evidence may suggests that Maca-GO, when taken in a right dose is acting as a toner of hormonal processes which may alleviate discomfort felt by women during early postmenopausal stage. However, there is also an indication, that due to strong sedative effect of Maca and strong anti-stress activity as observed in this study, when comparing scores which assessed intensity of symptoms experienced by the EPMW and expressed by their answering questions in the Greene’s Menopausal Index, Maca may induce calming effect which has different dynamics of action, compared to balancing or stimulating effect as may be interpreted by assessing changes in hormonal levels after Maca-GO administration. Muller (5, 6) also commented that, while low doses of Maca result in balancing effect on women, then, larger doses, 3g per day and above, may induce stimulation of hormonal secretion. Observations made in this pilot study justify further clinical research on use of Maca in postmenopausal women. This in order to assess effectiveness of Maca-GO as a non-hormonal therapeutic supplement which may help women to reduce discomfort associated with menopause as an alternative to HRT programs.

## CONCLUSION

In comparison to placebo, after both, two and eight months administration of Maca-GO capsules to EPMW, level of FSH significantly (*P*<0.05) decreased with a simultaneous significant (*P*<0.05) increase in the LH level. At the same time, levels of both E2 and PG, significantly (*P*<0.05) increased, however after eight months of Maca-GO treatment only, without significant effect after two month of similar treatment. There was a significant (*P*<0.05) placebo effect resulting in an increased PG level after one month administration of placebo capsules.

According to responses given by EPMW in related to menopausal symptoms specified in Green’s questionnaire, both, short- and long-term Maca-GO administration has substantially reduced feeling of discomfort associated with menopause. There was however, a distinctive positive placebo effect as judged by responses given in Greene’s questionnaire.

Based on results obtained in this study, it is reasonable to suggest that Maca-GO was acting as a toner of hormonal processes in EPMW as judged by decrease in FSH and increased LH secretion by pituitary gland, which resulted in stimulation of both ovarian hormones, E2 and PG, which in turn, induced a substantial reduction in discomfort felt by women during early postmenopausal stage.

Preliminary observations outlined in this paper, particularly different responses of women to a short- and long-term administration of Maca-GO to EPMW, justify further clinical study on effectiveness of Maca as a non-hormonal therapeutic supplement, which may help women to reduce discomfort associated with menopause as an alternative to HRT program.
